# Biochemical and structural basis of mercuric reductase, *Gbs*MerA, from *Gelidibacter salicanalis* PAMC21136

**DOI:** 10.1038/s41598-023-44968-w

**Published:** 2023-10-19

**Authors:** Bashu Dev Pardhe, Min Ju Lee, Jun Hyuck Lee, Hackwon Do, Tae-Jin Oh

**Affiliations:** 1 Department of Life Science and Biochemical Engineering, Graduate School, SunMoon University, Asan, 31460 Republic of Korea; 2 Research Unit of Cryogenic Novel Material, Korea Polar Research Institute, Incheon, 21990 Republic of Korea; 3 Department of Polar Sciences, University of Science and Technology, Incheon, 21990 Republic of Korea; 4 Genome-Based BioIT Convergence Institute, Asan, 31460 Republic of Korea; 5 Department of Pharmaceutical Engineering and Biotechnology, SunMoon University, Asan, 31460 Republic of Korea

**Keywords:** Biochemistry, Structural biology

## Abstract

Heavy metals, including mercury, are non-biodegradable and highly toxic to microorganisms even at low concentrations. Understanding the mechanisms underlying the environmental adaptability of microorganisms with Hg resistance holds promise for their use in Hg bioremediation. We characterized *Gbs*MerA, a mercury reductase belonging to the mercury-resistant operon of *Gelidibacter salicanalis* PAMC21136, and found its maximum activity of 474.7 µmol/min/mg in reducing Hg^+2^. In the presence of Ag and Mn, the enzyme exhibited moderate activity as 236.5 µmol/min/mg and 69 µmol/min/mg, respectively. *Gbs*MerA exhibited optimal activity at pH 7.0 and a temperature of 60 °C. Moreover, the crystal structure of *Gbs*MerA and structural comparison with homologues indicated that *Gbs*MerA contains residues, Tyr437´ and Asp47, which may be responsible for metal transfer at the *si*-face by providing a hydroxyl group (−OH) to abstract a proton from the thiol group of cysteine. The complex structure with NADPH indicated that Y174 in the *re*-face can change its side chain direction upon NADPH binding, indicating that Y174 may have a role as a gate for NADPH binding. Moreover, the heterologous host expressing *Gbs*MerA (pGbsMerA) is more resistant to Hg toxicity when compared to the host lacking *Gbs*MerA. Overall, this study provides a background for understanding the catalytic mechanism and Hg detoxification by *Gbs*MerA and suggests the application of genetically engineered *E. coli* strains for environmental Hg removal.

## Introduction

Heavy metals are naturally occurring elements found in various environments. These metals are typically present in trace amounts on Earth, but human activities such as mining, research, and industrial processes can introduce higher levels of heavy metals. Antarctica is not exempt from heavy metal contamination^1^. Heavy metals enter Antarctica through Long-range Atmospheric Transport (LRAT) from other continents, and their concentration levels rise due to anthropogenic activities, including research stations and other infrastructure^1^.

Over the decades, although the cold adaptation mechanisms of psychrophilic bacteria in Antarctica have been studied intensively, little attention has been given to the effects or mechanisms of heavy metal resistance or adaption in these bacteria^2–6^. Microorganisms found in soil and aquatic habitats are exposed to heavy metals such as copper(Cu), lead(Pb), and mercury(Hg)^6^. According to the report by Subhavana et al., while monitoring heavy metal contamination in Antarctica, the mercury levels in moss are approximately 66 ± 37 ng/g dry weight (dw), comparable to levels in other Antarctic locations, and have been the a major cause of heavy metal pollution in Antarctic habitats^7^. Atmospheric heavy metals, transported from lower latitudes, are non-biodegradable and toxic to microorganisms, with mercury (Hg) being highly toxic to most organisms, which can significantly alter microbial communities^8^. Additionally, chronic health effects in humans due to the inhalation of even low concentrations of mercury (0.7–42 µg/m^3^) have been reported^9^. Therefore, the study of environmental adaptability and coping systems for heavy metal resistance makes microorganisms potential candidates for heavy metal bioremediation^10^.

Studying gene sequences, flanking regions, and associated operons from microbial genome projects provides valuable insights into gene evolution. In this study, we applied this approach to investigate the mercuric reductase (MerA), the key enzyme within the mercury-resistant (*mer*) operon. Proteins encoded by the *mer* operon in microorganisms play a crucial role in Hg resistance and detoxification^11,12^. MerA (mercuric reductase) and MerB (organomercury lyase) are the central enzymes in the *mer* operon responsible for detoxifying inorganic (Mg(II)) and organic (e.g., methylmercury) mercury, respectively, in many bacteria thriving in Hg-contaminated environments^13,14^. For instance, metagenomic analysis of archaea and bacteria has revealed that homologs of MerA and MerB are present in 7.8 and 2.1% of genomes, respectively^14^.

MerAs are part of the disulfide oxidoreductase (DSOR) family^15^, which originated with the widespread oxygenation of Earth’s ecosystem that occurred after a Great Oxidation Event ~ 2.4 billion years ago^16^. Some hypotheses support the existence of Hg-resistant organisms in Hg-exposed geothermal and hydrothermal habitats^16,17^. Vetriani C et al. reported six out of eight mesophiles and four out of six moderate thermophiles (genus Alcanivorax) isolated from deep-sea hydrothermal vents displayed resistance to Hg(II) concentration > 10 µM^17^. On the other side, the comprehensive analysis of 272 individual *mer* operons by Boyd ES et al. provided that merA is a suitable biomarker to examine the diverse occurrence and function of *mer* operons for Hg detoxification in natural environments^18^.

*Gelidibacter* species have been found in various habitats and are of particular interest due to their ability to adapt to extreme environmental conditions^2–4^. For example, *Gelidibacter algens*, originating from Antarctica, grows at 0 °C and optimal growth temperature ranges from 15 to 25 °C; thus, serves as a model psychrophilic bacterium^3^, while the *Gelidibacter mesophilus* sp. from Mediterranean seawater can grow up to 37 °C, depending on the medium used^5^. Moreover, research on *Gelidibacter* and related species exploring their mechanisms on environmental adaptability and heavy metal bioremediation make them valuable subjects for studying how microorganisms cope with harsh surroundings^2–7^.

Therefore, in this study, genomic analysis of the psychrophilic bacterium *G. salicanalis* PAMC21136 from the soil of the Antarctic region was performed to identify *mer* operons. The structural and functional characterization of the key enzyme MerA from *G. salicanalis* (*Gbs*MerA) was performed. Furthermore, the molecular mechanism underlying mercuric detoxification by *Gbs*MerA was elucidated.

## Results and discussion

### *Gbs*MerA is part of the mercury-resistant operon

**Figure 1:**
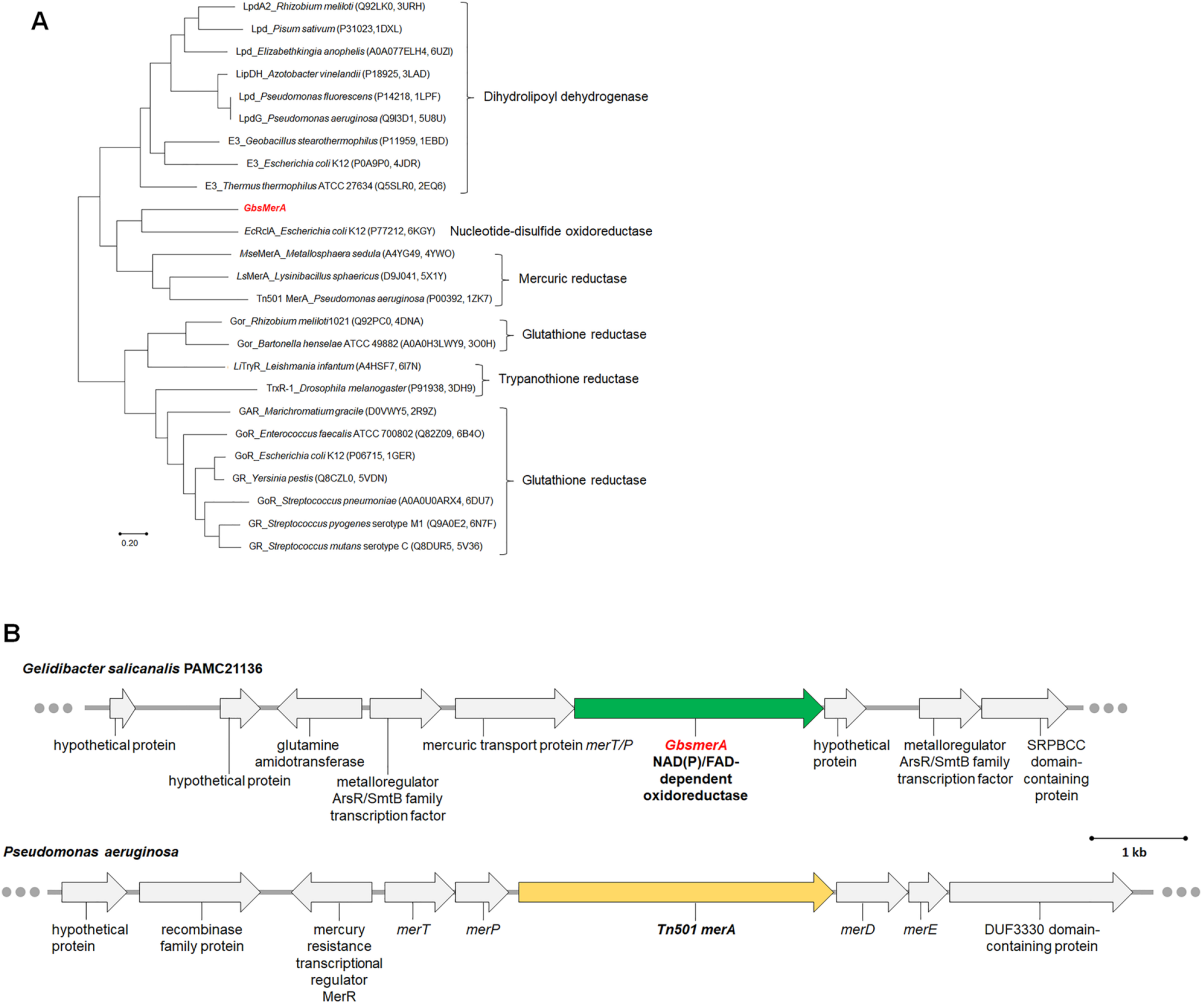
Bioinformatics analysis. (**A**) Phylogenetic analysis of *Gbs*MerA sequences. Homologs of *Gbs*MerA were obtained from various organisms by blasting the PDB database. In total, 25 protein sequences from the PDB database were aligned with *Gbs*MerA using an unrooted neighbor-joining tree by MEGA-X^36^. A consensus tree following 500 bootstrap replications is shown. The protein name, strain, UniProt ID, and PDB code were shown sequentially. (**B**) Scale model of gene organization in the genome of *Gelidibacter salicanalis* PAMC21136 and *Pseudomonas aeruginosa*.

To investigate the biological role of *Gbs*MerA, bioinformatics analysis of the *Gbs*MerA sequence and the genes flanking *Gbs*MerA was performed. Homologs of *Gbs*MerA were obtained from various organisms by using the BLAST search in the protein data bank (PDB) database^19^. Based on the phylogenetic tree, the reductases were divided into three groups. The group containing *Gbs*MerA also contains other mercuric reductases, namely *Mse*MerA from *Metallosphaera sedula*^20^, *Ls*MerA from *Lysinibacillus sphaericus*^21^, Tn501MerA from *Pseudomonas aeruginosa*^22^, and the nucleotide-disulfide oxidoreductase, *Ec*RclA, from *Escherichia coli* K12^23^. *Gbs*MerA is further intertwined within *Ec*RclA from *E. coli* K12 to form a separate clade (Fig. 1A). In addition, the genomic annotation of *G. salicanalis* indicated that *GbsmerA* is part of the *mer* operon comprising several types of genes similar to that of *P*. *aeruginosa* (Fig. 1B). The gene upstream of *GbsmerA* is MerT/P, which is responsible for Hg binding and/or transportation^24^. The gene downstream of *GbsmerA* encodes a hypothetical protein. Three genes are sequentially composed in the same direction without an intergenic gap, indicating that these genes are transcribed under the control of a single promoter^24^. The metalloregulator, ArsR, is coded upstream of *merT/P* in the same direction as the rest of the genes of the operon and is probably responsible for Hg sensing by regulating the transcription of the *mer* operon. These analyses indicated that *Gbs*MerA is a mercury reductase and is probably responsible for Hg detoxification in *G. salicanalis* PAMC21136, isolated from Antarctic soil as part of the Hg-resistant operon.

**Figure 2:**
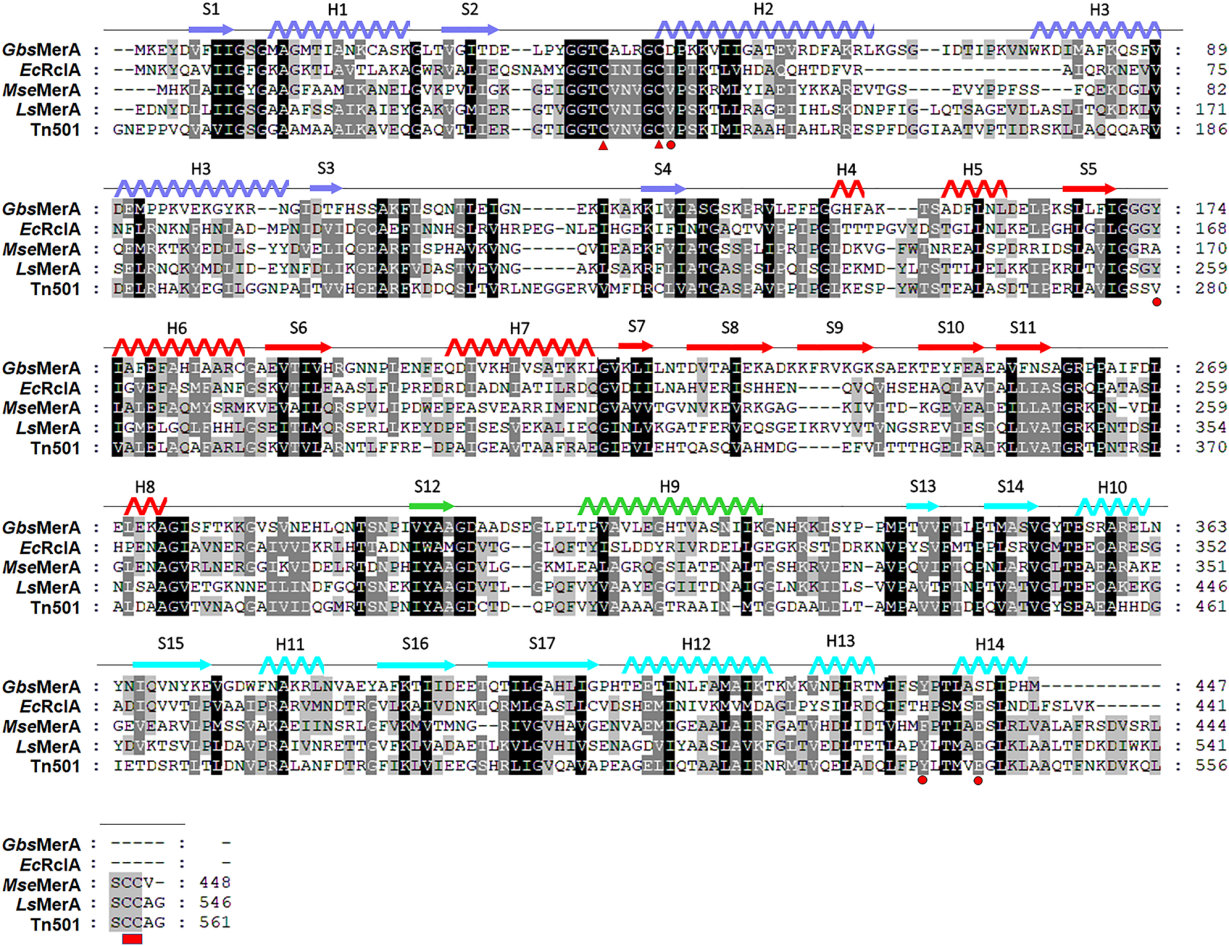
Multiple sequence alignment with other homologs, corresponding phylogenetic tree. Homologs of *Gbs*MerA were obtained from various organisms by blasting the PDB database. Mercuric reductases; *Mse*MerA from *Metallosphaera sedula*^20^, *Ls*MerA from *Lysinibacillus sphaericus*^21^, Tn501MerA from *Pseudomonas aeruginosa*^22^, and nucleotide-disulfide oxidoreductase, *Ec*RclA from *Escherichia coli* K12. The secondary structure of *Gbs*MerA with the same color codes shown in Fig. 4A is indicated on the top of the sequences. The Cys42-Cys46 pair is indicated with a red triangle. The Asp47, Tyr174, and Tyr437' are marked with red circles at the bottom of the alignment. The Cys–Cys motif in the C-terminal end loop was indicated with red rectangle. Consensus amino acid residues conserved in > 60% of proteins are indicated by light grey background, whereas residues conserved in > 80% and 100% of proteins are indicated with grey and black background, respectively.

Moreover, multiple sequence alignments with homologs showed that *Gbs*MerA has unique sequential features that differ from those of other MerAs (Fig. 2). Previous studies have indicated that the N-terminal domain (NmerA) of MerA is responsible for recruiting Hg^2+^ and transferring it to flexible C-terminal segments containing a pair of cysteines^22,25^. Moreover, the C-terminal cysteine pair undergoes a conformational change to deliver Hg^2+^ to the active site of the opposing monomer^25^. However, the N-terminal domain and C-terminal segments, including the Cys–Cys motif, were absent in *Gbs*MerA, indicating that *Gbs*MerA potentially has a mechanism different from that of MerAs, except *Ec*RclA (Fig. 2 and Supplemental Table S1).

### Purification and biochemical properties of *Gbs*MerA

To understand the reductase activity and characteristics, *Gbs*MerA was purified, and its biochemical properties were evaluated. *GbsmerA* of *G. salicanalis* was amplified and cloned into the pET32a( +) vector under the control of the T7 promoter. After the transformation of *E.coli* BL21 with the cloned vector, *Gbs*MerA was overexpressed and successfully purified using Ni–NTA purification. The purity of the recombinant *Gbs*MerA was higher than 95% when analyzed using sodium dodecyl sulfate (SDS)-densitometry (Supplemental Figure S1A). The recombinant protein was cleaved from the vector tag and further purified *Gbs*MerA by size-exclusion chromatography (SEC) (Supplemental Figure S1B). Purified *Gbs*MerA exhibited a peak at 376 nm, a Soret peak was observed at 462 nm, and a deep trough was observed at 404 nm in its oxidized form, which is a spectral feature of other flavoproteins, including MerAs (Supplemental Figure S1C)^26,27^.

**Figure 3:**
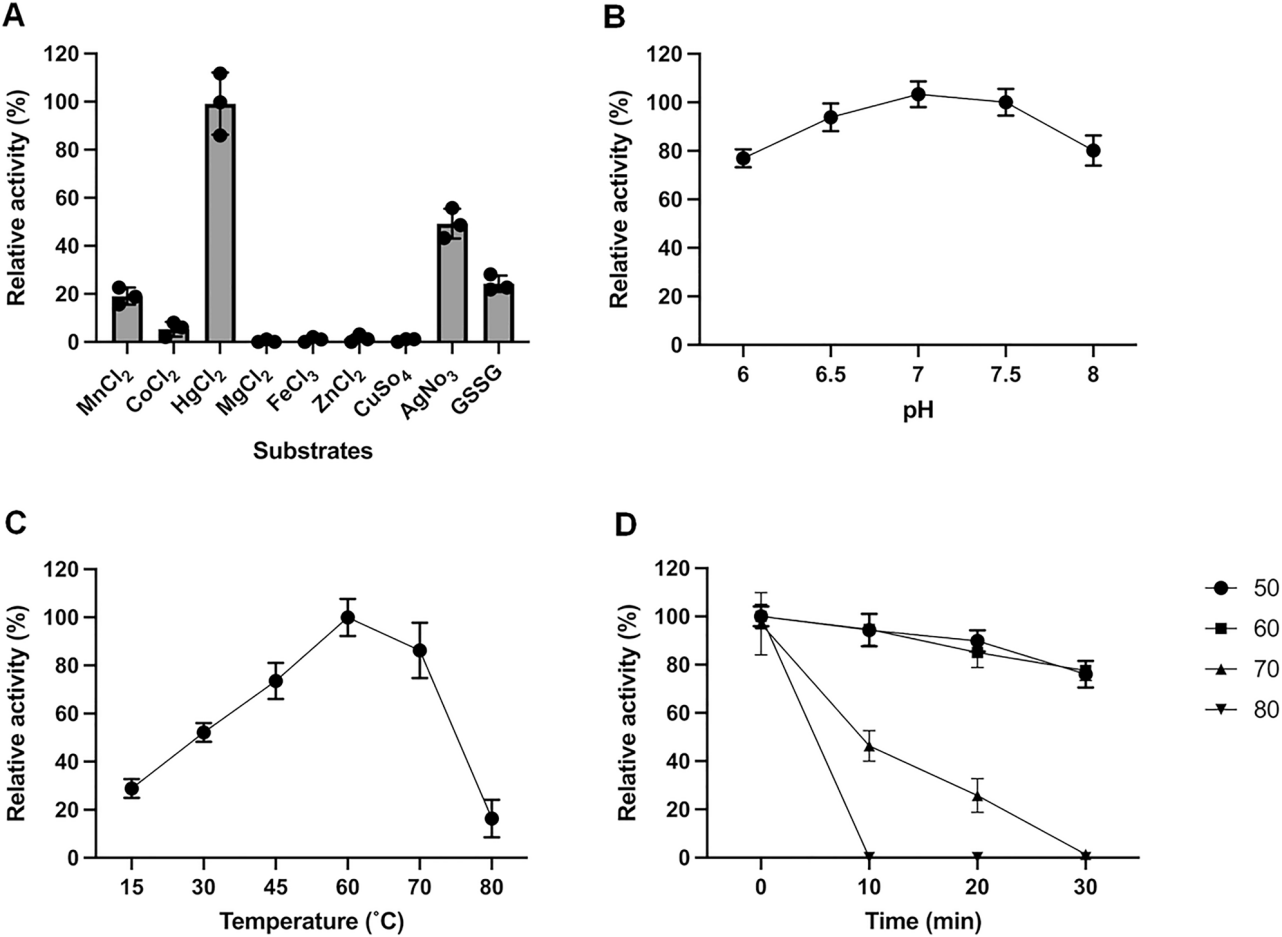
Biochemical properties of *Gbs*MerA. (**A**) Relative activity of *Gbs*MerA with different substrates. The reaction was carried out in 50 mM potassium phosphate buffer containing 12.8 µg enzyme. The reaction was initiated by 100 µM NADPH and the rate of oxidation was measured for 3 min. (**B**) The optimal pH was determined at the range of pH 6 to 8. (**C**) The temperature-dependent activity was measured at a temperature range from 20 to 80 °C. (**D**) The stability of mercuric reductase was determined at a temperature range from 50 to 80 °C. The activity was measured at different time intervals.

Previous studies on MerAs indicated that metal reductases catalyze the reduction of a wide range of substrates^21^. Therefore, the specific activity of *Gbs*MerA was measured using various metals (MnCl_2_, CoCl_2_, MgCl_2_, FeCl_3_, ZnCl_2_, CuSO_4_, and AgNO_3_, and HgCl_2)_. Consistent with the bioinformatics findings, the experimental results showed the highest oxidation of NADPH with Hg ions (inorganic Hg) by *Gbs*MerA, as measured by the specific activity of 474.7 µmol/min/mg (Table 1). The oxidation activity decreased to 50% in the presence of Ag^+^ compared with that in the presence of Hg^+2^. Approximately 25% of activity was measured in the presence of Mn ions or GSSG. The trivial or undetectable activity was measured upon the addition of Co, Mn, Fe, Zn, or Cu ions (Fig. 3A). Thus, this analysis confirmed that *Gbs*MerA is a mercury reductase (EC 1.16.1.1) that is probably responsible for Hg detoxification in *G. salicanalis* as part of the Hg-resistant operon.

**Table 1:** Specific activity *Gbs*MerA in the presence of various substrates.

Substrates	Specific activity (µmol/min/mg)
HgCl_2_	474.7
AgNo_3_	236.5
GSSG	97.3
MnCl_2_	69.0

The activity was measured by checking the amount of oxidized NADPH. The reaction was initiated by 100 µM NADPH and the rate of oxidation was measured for 3 min. As high concentrations of HgCl_2_ and AgNo_3_ cause protein precipitation, only 50 µM concentration was used for these substrates.

Furthermore, the pH for *Gbs*MerA optimal activity was investigated over a pH range from 6.0 to 8.0. *Gbs*MerA showed maximal activity at pH 7.0, and there was no significant decline in activity at pH 6.0 or 8.0 (Fig. 3B). Furthermore, *Gbs*MerA exhibited the maximum activity of 474.7 µmol/min/mg in reducing Hg^+2^ at a temperature of 60 °C and retained more than 80% of enzyme activity even after 30 min incubation (Fig. 3C and D). However, *Gbs*MerA activity was completely lost within 10 min at 80 °C.

### Structural determination and overall structure of *Gbs*MerA

We performed X-ray crystallography and structural characterization to gain mechanistic insights into *Gbs*MerA structures. Crystals of *Gbs*MerA (35 mg·mL^−1^) were obtained in drops of a 1:1 mixture of protein and crystallization buffer (1.6 M sodium phosphate monobasic/0.4 M potassium phosphate dibasic, 0.1 M sodium phosphate dibasic/citric acid [pH 4.2]). The initial structure was determined by molecular replacement using the model generated by AlphaFold^28^. After interactive refinement of the coordinate, the structure was refined with a Rwork of 20.2% and a Rfree of 24.2% at 2.6Å (Supplemental Table S2). Two *Gbs*MerA monomers, forming a homodimer with two-fold symmetry, were found in the asymmetric unit. Furthermore, we determined the *Gbs*MerA–NADPH complex structure. The crystals of the complex were obtained under the crystallization conditions of 10% w/v PEG 4000, 20% v/v glycerol, 0.12 M of ethylene glycol, and 0.1 M bicine/Trizma pH8.5. The X-ray diffraction dataset of the complex was collected, processed, and determined similar to that of *Gbs*MerA.

**Figure 4:**
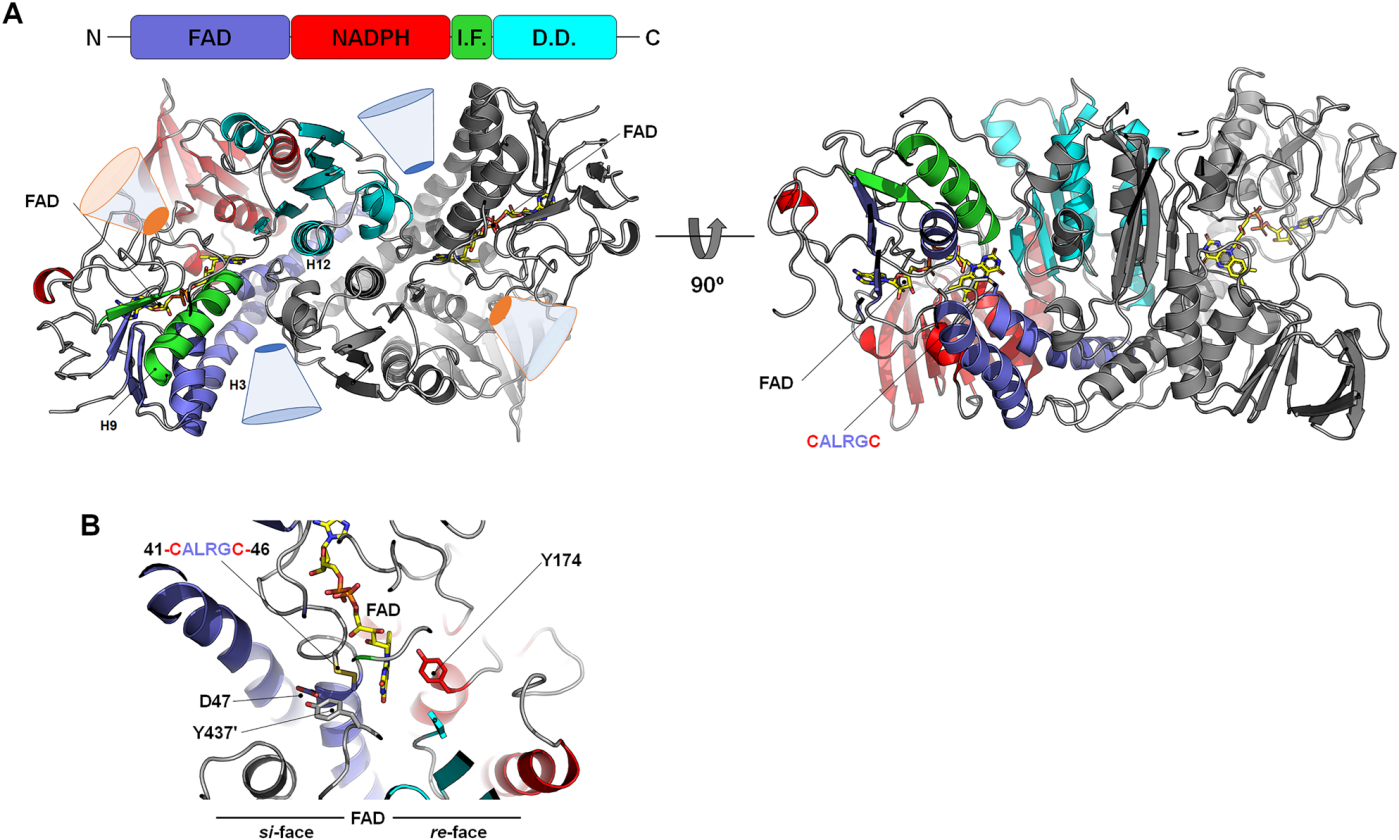
Crystal structure of *Gbs*MerA. (**A**) The overall structure of *Gbs*MerA consists of a FAD-binding domain (FAD, residues 1–146), an NADPH-binding domain (NADPH, residues 147–275), an interface domain (IF, residues 276–340), and dimerization domain (DD, residues 341–447). (**B**) Close-up view of the *Gbs*MerA active site. The active site can be divided based on the isoalloxazine ring of FAD. Tyr437´ and Asp47 are located proximate to the Cys–Cys pair at the *si*-face and a side chain of Tyr174 points perpendicularly to the flavin ring. The residue sticks are colored with the same color of its domain shown in Figure A. FAD is shown in yellow sticks.

As seen in the structures of thioredoxin reductase (high Mr), trypanothione reductase, glutathione reductase, glutathione amide reductase, and dihydrolipoamide dehydrogenase, the overall structure of *Gbs*MerA also comprises an FAD-binding domain (residues 1–146), an NADPH-binding domain (residues 147–275), an interface domain (residues 276–340), and a dimerization domain (residues 341–447)^29^ (Fig. 4A). Whole residues of the two monomers in the asymmetric unit could be built, except for regions 112–115 and 120–126 of chain B because of the unclear electron density. Non-covalently bound FAD is located in the central region of the FAD-binding domain, with two hydrophobic interactions, 16 hydrogen bonds, two water bridges, and π-stacking interactions. An unambiguous redox-active disulfide bond between Cys41 and Cys46 was found in the opposite NADPH-binding domain (Fig. 4B).

### Structural feature of *Gbs*MerA and comparison with MerAs

Structural homologs of *Gbs*MerA have been obtained from various organisms using the DALI server^30^ (Supplemental Table S1). Similar to the phylogenetic tree, the results showed that *Ls*MerA from *L. sphaericus* (PDB code:5X1Y; Z-score = 45.8), *Mse*MerA from *M. sedula* (4YWO; Z-score = 44.7), *Ec*RclA from *E. coli* K12 (6KGY; Z-score = 44.0), and Tn501MerA_*P. aeruginosa* (1ZK7; Z-score = 43.2) had structures most similar to *Gbs*MerA. Based on the analysis of the phylogenetic tree and a search for structural homologues, *Ec*RclA closely resembles *Gbs*MerA and both *Gbs*MerA and *Ec*RclA lack the Cys–Cys motif at the C-terminal end loop for metal ion binding, implying that two proteins could share the reduction mechanism.

Although the structural fold and FAD- and NADPH-binding modes of *Gbs*MerA were similar to those of MerAs, the residual composition at the active site was different. *Gbs*MerA has two pathways that connect molecular surfaces to the *si*- and *re*-faces of the isoalloxazine ring in FAD (Fig. 4B) (Supplemental Figure S2). In the *si*-face channel, two cysteines (Cys41 and Cys46) from the C*XXXX*C motif form a disulfide bond, and Asp47, Thr312, and Tyr437´ from the neighboring chain surround the disulfide bond and seem to be involved in the transfer of Hg^2+^ or the reduction of disulfide-bonded substrates^20^. In *Ec*RclA, His426´ and Glu431´ of the H*XXXX*E motif (known as the His-Glu pair in Group 1 FDR Enzymes)^31^ receive protons from two cysteines of the C-terminus and transfer them to O_2_^23^. However, this His–Glu pair is substituted by Tyr437´ and Ser442´ in the corresponding region in the *Gbs*MerA structure. In Tn501MerA, Tyr441´, which corresponds to Tyr437´ of *Gbs*MerA, is responsible for metal transfer by coordinating the metal with the hydroxyl group. Tyr100 in Tn501MerA is assumed to be important for transferring metals along with Tyr441^22^. However, Tyr100 has been replaced with Val96 in *Gbs*MerA. Thus, instead of His426 in *Ec*RclA, Tyr437 of *Gbs*MerA could donate a hydroxyl group (–OH) to abstract a proton from the thiol group of cysteine, resulting in cysteine deprotonation.

**Figure 5:**
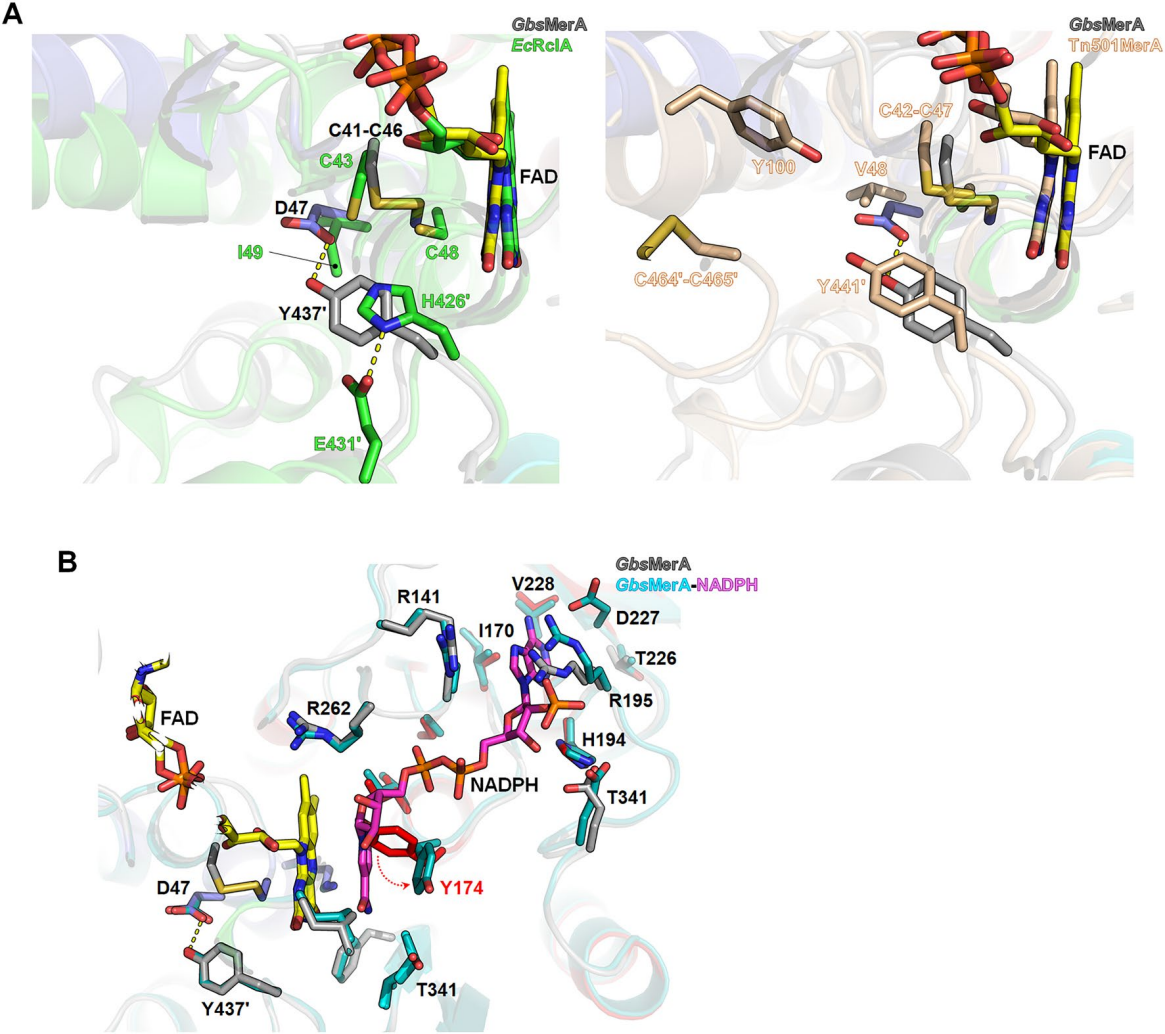
Structural comparison of GbsMerA with homologs. (**A**) The *si*-face of *Gbs*MerA (depicted in grey) is compared with *Ec*RclA (shown in green) on the left panel and Tn501MerA (wheat color) on the right panel. The FAD molecules are represented with the same color code as the respective proteins. The residues surrounding the two cysteines are illustrated as sticks. (**B**) Comparison between *Gbs*MerA and *Gbs*MerA–NADPH complex. The *re*-face of *Gbs*MerA is depicted with conserved residues in the NADPH binding site. The conformational change of the side chain of Tyr174 upon NADPH binding was indicated with a red arrow.

To investigate the role of Tyr437 in *Gbs*MerA metal transfer, we generated Y437F mutant. The relative activity showed that Y437F completely lost its activity, indicating that the hydroxyl group of Tyr437 is important for metal coordination during transfer (Supplemental Figure S3). Based on this observation, Tyr437´ along with Asp47 at the *si*-face in *Gbs*MerA may be involved in the metal transfer, and *Gbs*MerA has an alternative metal binding strategy for Hg^2+^ ions (Fig. 5A). Although this proposed reaction is indirectly proven by experiment with Y437F mutant, we anticipate that additional research is needed to fully elucidate the complete reactions.

The *re*-face channel exhibits a concave and funnel-like structure located at the interface of the NADPH-binding and dimerization domains. Residues at the NADPH-binding site are conserved, similar to those found in other MerAs, and generate positively charged surfaces. These residues included Arg141, His194, Arg195, and the nitrogen atoms of glycines (G171, and G173) (Fig. 5B and Supplemental Figure S2). Structural comparison between the FAD- and FAD–NADPH-bound forms indicated that these residues were not changed upon NADPH binding, except for Tyr174 and Arg195. Arg195, which is located at the edge of the central sheet of the NAPDH domain, was tilted toward the solvent area in the NADPH complex (Fig. 5B). Another residual change observed upon NADPH binding was Tyr174. Without NADPH, the side chain of Tyr174 points to the isoalloxazine ring of FAD by forming T-shaped configuration. However, in the NADPH complex, the side chain of Tyr174 was flipped opposite to the isoalloxazine ring of FAD by 6.5 Å (OH group). The Tyr174 residue was replaced with the nicotinamide ring of NADPH. Therefore, the isoalloxazine ring of FAD, nicotinamide ring of NAPDH, and Tyr174 are aligned and stacked with cation–π interaction (Fig. 5B and Supplemental Figure S2). Mutation of Tyr174 to Phe showed decreased activity by 50%, indicating that the interaction of Tyr174 with the isoalloxazine ring of FAD through the hydroxyl group of tyrosine may be important for controlling the NADPH binding. Notably, the corresponding tyrosine residue of Tyr174 is strictly conserved in glutathione reductase and thioredoxin reductase^32^. Similarly, previous studies on glutathione reductase indicated that this tyrosine serves as a gate for the binding of NADPH and plays a role in flavin fluorescence depolarization^33,34^. Based on the highly identical ligand-binding and geometric features of the *re*-face of glutathione reductase, a similar mechanism is expected for *Gbs*MerA.

### Hg detoxification by *Gbs*MerA

**Figure 6:**
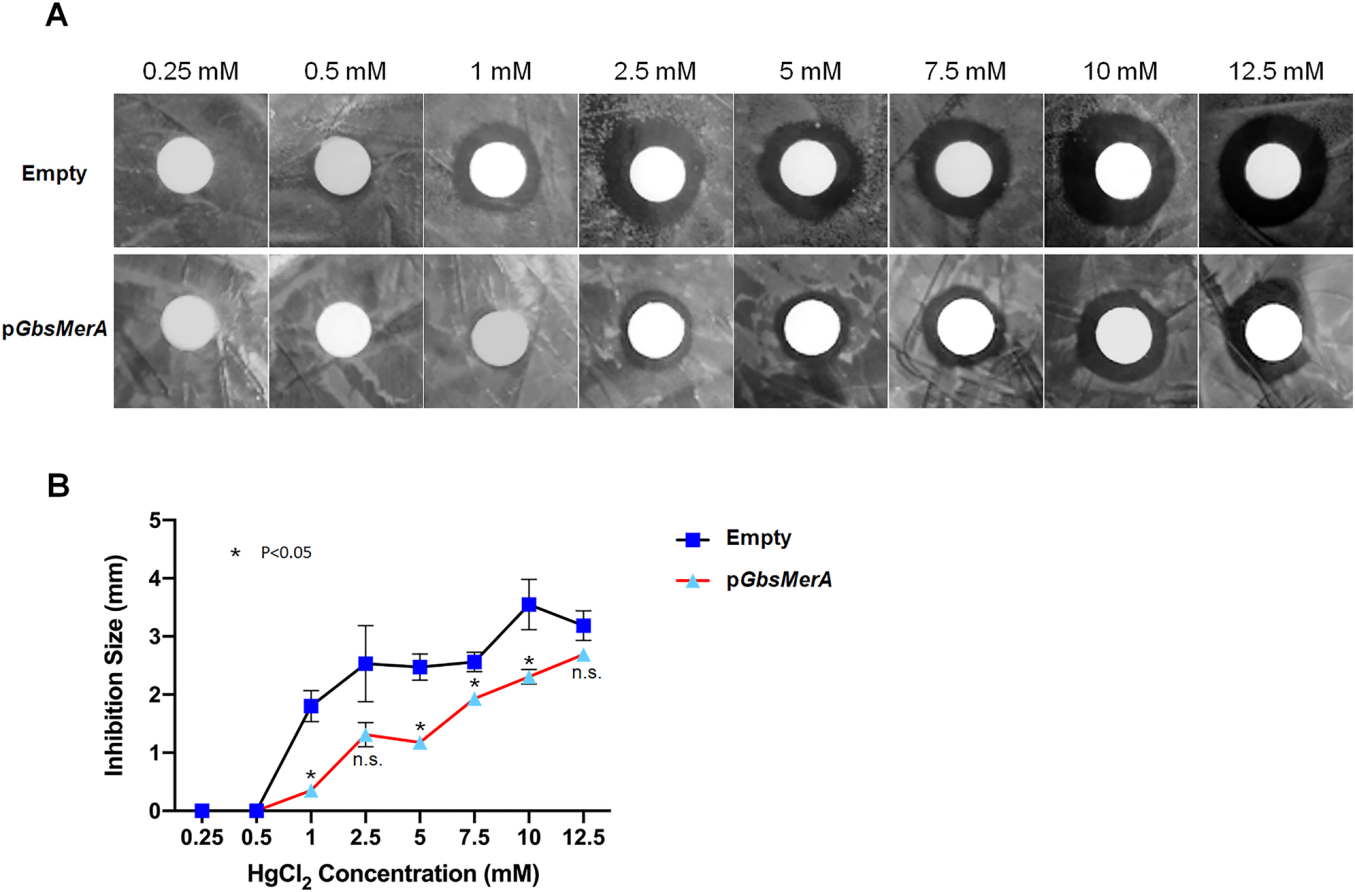
Determination of the minimum inhibitory concentration (MIC) for HgCl_2_. (**A**) Luria–Bertani agar plate inoculated with *E. coli* transformant containing *Gbs*MerA in pET32. (**B**) Inhibition zone size for each experimental group. Inhibition size from the paper disc was measured three times and averaged at each concentration of HgCl_2_. Inhibition LB-agar plate inoculated with *E. coli* transformant containing pET32. In total, 4 µL of increasing concentration of HgCl_2_ (0.25–10 mM) was applied over the 6 mm paper disc placed on an LB-agar plate supplemented with 50 µg/mL ampicillin and 0.5 mM isopropyl β-d-thiogalactoside. The plates were incubated for 24 h at 37 °C. The radii of the clear zones are a measure of the toxic effect of HgCl_2_ on bacterial growth. The paired t test analysis was conducted using GraphPad Prism version 9.0. The data is presented as mean ± SD. “ns” signifies non-significance (*p* value ≥ 0.05), while an asterisk indicates a significant difference (*p* value < 0.05).

Microorganisms utilize mercuric reductase to reduce toxic ionic Hg (Hg^2+^) to its less toxic, volatile, and elemental form (Hg^0^). To investigate the functional role of *Gbs*MerA, we evaluated its Hg^2+^-scavenging effect by comparing the strain with an empty vector (pET32/BL21 [DE3]) and a *Gbs*MerA-producing strain (p*GbsMerA*-pET32/BL21 [DE3]). The strains were spread on the agar plate supplemented with 50 µg/mL ampicillin and 0.5 mM IPTG and applied series of HgCl_2_ concentrations on the filter paper. As shown in the figure (Fig. 6A), the heterologous host containing *Gbs*MerA (p*GbsMerA*) showed a significant decrease in the clearance zone compared with the host without *Gbs*MerA (empty) (Fig. 6B). This result indicated that *Gbs*MerA has detoxification properties and can remove ionic Hg, making it more resistant to Hg toxicity.

## Conclusions

In this study, we identified and characterized mercuric reductase (*Gbs*MerA), which is coded as a component of the mercury resistance (*mer*) operon in *G. salicanalis* isolated from Antarctic soil. Structural analysis and mutagenesis experiments reveal that the hydroxyl group of Tyr437 at the *si*-face is important for metal coordination during transfer. Furthermore, structural comparison between the FAD and FAD_NADPH complex form indicates that the isoalloxazine ring of FAD through the hydroxyl group of Tyr174 at the *re*-face may be essential for controlling the NADPH binding. Functional characterization reveals that *Gbs*MerA has the ability to reduce ionic mercury. More importantly, functional characterization using a heterologous host (BL21) expressing *Gbs*MerA is potent for the detoxification of Hg^2+^. This study not only provides structural insight into the reductase mechanism but also underscores the promising role of microorganisms in mercury detoxification within industrial contexts.

Based on the analysis of the phylogenetic tree and a search for structural homologues, *Ec*RclA closely resembles *Gbs*MerA and both *Gbs*MerA and *Ec*RclA lack the Cys–Cys motif at the C-terminal end loop for metal ion binding, implying a shared reduction mechanism. In the case of *Ec*RclA, it contains the His426–Glu431 pair within the HXXXXE motif on the *si*-face, which is known to facilitate the deprotonation of Cys43^25^. However, in the *Gbs*MerA structure, this His–Glu pair is substituted with Tyr437–Ser442 in the corresponding region in the *Gbs*MerA structure. Instead of His426 in *Ec*RclA, Tyr437 of *Gbs*MerA could donate a hydroxyl group (–OH) to abstract a proton from the thiol group of cysteine, resulting in cysteine deprotonation. Although this proposed reaction is indirectly proven by experiment with Y437F mutant, we anticipate that additional research is needed to fully elucidate the complete reactions.

## Methods

### Chemicals and reagents

T4 DNA ligase, DNA polymerase, and dNTPs were procured from Takara Bio (Shiga, Japan). Hydrogen peroxide (H_2_O_2_), nicotinamide adenine dinucleotide phosphate (NADPH), oxidized glutathione (GSSG), manganese(II) chloride (MnCl_2_), cobalt chloride (CoCl_2_), mercuric chloride (HgCl_2_), magnesium chloride (MgCl_2_), ferric chloride (FeCl_3_), zinc chloride (ZnCl_2_), copper sulfate (CuSO_4_), and silver nitrate (AgNO_3_) were procured from Sigma-Aldrich (Yongin, Korea). Isopropyl-1-thio-β-D-galactopyranoside (IPTG), ampicillin, and kanamycin were purchased from Duchefa Bohemie (Seoul, Korea). Restriction enzymes were purchased from Takara Clontech (Shiga, Japan).

### Cloning and purification of GbsMerA

*GbsmerA* encoding 449 amino acids obtained from *G. salicanalis* PAMC21136 was amplified using the specific PCR forward primer 5′-GGATCCATGAAAGAATATGATGTT-3′ (BamHI) and reverse primer 5′-CTCGAGTTAAAGCATATGCGGTAT-3′ (XhoI). The purified PCR product was cloned into the pMD20-T vector using *Escherichia coli* XL1-Blue, selected by blue–white screening, and the nucleotide sequences were confirmed by automated sequencing (Macrogen, Suwon, Korea). The confirmed genes were ligated into pET32a( +) and the construct was transformed into *E. coli* XL1-Blue. The construct was plated on Luria–Bertani (LB) agar containing 100 μg/mL ampicillin. The amplified construct DNA encoding the N-terminal His6-tag protein under the control of a T7 promoter was isolated and transformed into chemically competent BL21 cells for overexpression and plated on LB agar containing 100 μg/mL ampicillin. Three milliliters of seed culture was grown for 4 h from a single colony under stress (100 μg/mL of antibiotics) as follows: 0.3 mL seed culture was added to 100 mL of LB medium supplemented with 100 μg/mL of antibiotics and incubated at an orbital shaker (200 rpm) at 37 °C until the cell density was about 0.6 at an OD of 600 nm. Induction was performed with 0.4 mM of Isopropyl β-D-1-thiogalactopyranoside (IPTG) and the culture was incubated for 24 h at 20 °C for protein synthesis. Cell pellets were harvested by centrifugation (3500 rpm) for 30 min at 4 °C and washed twice with 50 mM potassium phosphate buffer (pH 7.4). The harvested cell pellets were suspended in potassium phosphate buffer (pH 7.4) and lysed by ultrasonication (Vibra-Cell VCX400). The soluble protein-containing fraction was separated by centrifugation at 24,650 × g for 20 min at 4 °C and purified by Ni^2+^ affinity chromatography with the use of TALON His-tag. Resin-bound proteins were eluted by using elution potassium buffer (7.4) containing 10% glycerol, 100 mM NaCl, and different concentration gradient of imidazole (10, 100, and 250 mM). The purity of the protein in all fractions was confirmed by performing SDS–polyacrylamide gel electrophoresis (SDS–PAGE) and the purified fraction was concentrated by ultrafiltration using Amicon centrifugal filters with a cut-off molecular weight of 50 kDa. Further, *Gbs*MerA was loaded into the size exclusion chromatography (SEC) column after enterokinase cleavage. Factions were collected for the crystallization. Superdex 200 10/300 GL column connected with ÄKTA Avant system (Cytiva, Marlborough, MA, USA) was used.

### Protein estimation and characterization

Protein concentration was measured using the Bradford method by using bovine serum albumin as the standard^35^. NADPH oxidation was performed in 50 mM potassium phosphate buffer containing 12.8 µg of the enzyme in the presence of different substrates (GSSG, MnCl_2_, CoCl_2_, HgCl_2_, MgCl_2_, FeCl_3_, ZnCl_2_, CuSO_4_, and AgNO_3_). The reaction was initiated by using 0.1 mM NADPH and the rate of oxidation was measured for 3 min. A blank was prepared under the same conditions except that the substrates were added, the background of NADPH oxidation was subtracted, and the enzymatic activity was calculated. One unit of enzyme activity was defined as the amount of substrate that oxidizes 1 µM of NADPH per minute. The optimum pH of *Gbs*MerA was determined over a pH range of 6.0–8.0 using 50 mM potassium phosphate buffer. Temperature-dependent activity was measured at pH 7.0 from 20 to 80 °C. Residual activity was measured at 40 to 80 °C. In total, 12.8 µg of the enzyme and 50 µM HgCl_2_ in buffer were incubated at different time intervals (0, 10, 20, and 30 min) at different temperatures, the reaction was initiated by adding 0.1 mM NADPH, and the oxidation rate was measured. The highest residual activity at the defined temperature was mentioned as 100%. Purified *Gbs*MerA (12.8 µg) was oxidized with 50 µM HgCl_2_ aerobically in 50 mM potassium phosphate buffer at 25 °C and UV–visible spectra were recorded from 300 to 600 nm for performing spectral analysis. All the samples were scanned using a Biochrom Libra S35PC UV/visible spectrophotometer (Cambridge, UK).

### Bioinformatics analysis

Orthologs of *Gbs*MerA were identified by BLAST sequence analysis using Protein Data Bank (PDB) and UniProtKB/Swiss-Prot (Swissprot) databases from National Centre for Biotechnology Information (https://blast.ncbi.nlm.nih.gov). Identical and predicted amino acid sequences from different species were aligned using the CLUSTAL W program (https://www.ebi.ac.uk/Tools/msa/clustalo/). The phylogenetic tree was developed by Mega X using Neighbor-Joining algorithms^36^. Bootstrap values were calculated based on 500 replicates for confidentiality.

### Crystallization and structure determination

*Gbs*MerA protein crystals were developed using the sitting drop vapor diffusion method at 22 °C for two days. Diffraction-quality crystals of *Gbs*MerA were obtained after initial crystallization screening and optimization of the crystallization conditions. All crystals were stabilized in a crystallization solution without a cryoprotectant before flash freezing in a liquid nitrogen stream for data collection at − 173 °C. All X-ray diffraction data (wavelength = 0.979 Å) were collected at the 5C beamline of Pohang Accelerator Laboratory (PAL, Korea). An HKL3000^37^ was used to index, integrate, and scale the diffraction data. The structure of *Gbs*MerA was determined by molecular replacement and iterative manual model building, and refined using COOT^38^ and REFMAC5^39^, and Phenix-refine^40^. A simple flat bulk solvent model implemented in the program REFMAC was applied for bulk solvent correction^41^, and 5% of the reflections were selected for Rfree calculations^42^. Water was added to the *Gbs*MerA model based on the default parameters of Phenix. The crystallographic and regimen statistics are summarized in Supplemental Table S2.

### Site-directed mutagenesis

Mutagenesis was performed using the EZchange™ site-directed mutagenesis kit from enzynomics, with some modifications. PCR mixture contained 5 μL of 10 × reaction buffer, 10 pmol/μL of forward and reverse primers (1.25 μL each), 4 μL of dNTP mixture (2 mM), 50 ng (1.25 μL) of the template pET32a containing *GbsmerA* (p*Gbs*MerA), and ddH_2_O was added to obtain a final volume of 50 μL. *n*pfu-Forte DNA polymerase (1 μL; 2.5 units/μL) was added to each reaction mixture. PCR was performed according to the manufacturer’s protocol. Elongation was performed for 7 min at 72 °C in each cycle for a total of 25 cycles. All primers were designed to anneal at 55 °C and are listed in Supplemental Table S3. The PCR product (10 μL) digested using the EZ-MIX restriction enzyme at 37 °C for 1 h was used for heat shock transformation into 50 μL aliquots of NEB5α-competent *E. coli* cells. SOC broth (50 μL) was added to the transformed mixture and incubated at 37 °C for 1 h with shaking at (225–250) rpm. NEB5α cells were plated on Luria–Bertani broth (LB, Miller) agar containing 25 μg/mL of ampicillin and incubated at 37 °C overnight. Colonies for each mutagenic reaction were picked, cultured in LB broth supplemented with 25 μg/mL of ampicillin at 37 °C, and shaken at 220 rpm for 10 h, followed by plasmid isolation, digestion, and confirmation by automated sequencing (Macrogen, Korea). Confirmed mutants were transformed into *E. coli* BL21 (DE3) cells and the overexpressed and purified proteins were used to determine the NADPH oxidation rate.

### Disk diffusion assay

The survival rates of the strain with an empty vector (pET32/BL21 [DE3]) and the *Gbs*MerA-producing strain (p*GbsMerA*-pET32/BL21[DE3]) were compared. LB-agar plates supplemented with 50 µg/mL of ampicillin and 0.5 mM isopropyl β-d-thiogalactoside were inoculated with the strains containing an empty vector and *Gbs*MerA separately. An increasing concentration of HgCl_2_ (4 µL; 0.25 to 12.5 mM) was applied over a 6-mm paper disc placed on each LB agar plate. The plates were then incubated for 24 h at 37 °C. The radii of clear zones were considered a measure of the toxic effects of HgCl_2_ on bacterial growth. The experiments were performed in triplicates simultaneously at the same time and under the same conditions.

### Supplementary Information

Supplementary Information.

## Data Availability

Protein Data Bank accession numbers: The final refined coordinates and structural factors of *Gbs*MerA with FAD and *Gbs*MerA with FAD and NADPH were deposited in the RCSB Protein Data Bank under the accession codes 8K40 and 8K41, respectively.
