# Supplementary material for: Biochemical and structural basis of mercuric reductase, GbsMerA, from Gelidibacter salicanalis PAMC21136
Source: Sci Rep. 2023 Oct 19;13:17854. doi: 10.1038/s41598-023-44968-w (PMC10587081; doi:10.1038/s41598-023-44968-w)
Supplement: Supplementary file 1 — Supplementary Information. [file 41598_2023_44968_MOESM1_ESM.docx]

Biochemical and structural basis of mercuric reductase, *Gbs*MerA, from *Gelidibacter salicanalis* PAMC21136

**Supporting Information**

**Index**

**Supplemental Table S1.** Pairwise sequence identity of metal reductases

**Supplemental Table S2.** Primers used for site-directed mutagenesis

**Supplemental Table S3.** X-ray diffraction data collection and refinement statistics

**Supplemental Figure S1.** Purification of *Gbs*MerA

**Supplemental Figure S2.** Stereo view of the FAD binding site in the *Gbs*MerA monomer

**Supplemental Figure S3.** Comparison of the activity of *Gbs*MerA with mutants

**Supplemental Table S1.** Pairwise sequence identity of metal reductases.

|  | GbsMerA | EcRclA | MseMerA | LsMerA | Tn501 |
| --- | --- | --- | --- | --- | --- |
| GbsMerA | 100  100 |  |  |  |  |
| EcRclA | 28.4  48.3 | 100  100 |  |  |  |
| MseMerA | 26.8  45.5 | 27.0  49.3 | 100  100 |  |  |
| LsMerA | 24.0  37.3 | 25.8  45.3 | 32.6  49.9 | 100  100 |  |
| Tn501 | 22.3  36.8 | 24.5  40.1 | 30.7  44.6 | 41.6  59.3 | 100  100 |

The Web-based EMBOSS Needle ^1^ was used for pairwise alignment. Percentages of identity and similarity for each pair of enzymes are shown in the upper and lower halves of each box, respectively. The gap opening and gap extension penalties were set to 10 and 0.5, respectively.

**Supplemental Table S2.** X-ray diffraction data collection and refinement statistics.

| Data set | *Gbs*MerA with FAD | *Gbs*MerA with FAD, NADPH |
| --- | --- | --- |
| X-ray source | BL-5C beamline | BL-5C beamline |
| Space group | *P*2_1_2_1_2_1_ | *P*3_1_21 |
| Unit-cell parameters (Å, °) | a = 78.19, b = 105.82, c = 126.82, α = β = γ = 90 | a = b = 102.33, c = 108.70, α = β = 90.00, γ = 120 |
| Wavelength (Å) | 0.9794 | 0.9794 |
| Resolution (Å) | 29.38–2.60 (2.72–2.60) | 29.57–2.39 (2.48–2.39) |
| Total reflections | 435,278 (55,692) | 525,372 (53,853) |
| Unique reflections | 33,032 (3,967) | 26,169 (2,695) |
| Average I/σ (I) | 15.5 (2.0) | 27.8 (2.7) |
| *R*_merge_^a^ | 0.11 (1.60) | 0.09 (1.14) |
| Redundancy | 13.2 (14.0) | 20.1 (20.0) |
| Completeness (%) | 99.9 (99.9) | 99.0 (98.3) |
| Refinement |  |  |
| Resolution range (Å) | 29.38–2.60 (2.67–2.60) | 29.59–2.39 (2.45–2.39) |
| No. of reflections of working set | 33,022 (2,172) | 24,874 (1,785) |
| No. of reflections of test set | 2,000 (139) | 1,295 (94) |
| No. of amino acid residues | 6,904 | 3,349 |
| No. of water molecules | 33 | 13 |
| *R*_cryst_^b^ | 0.20 (0.26) | 0.20 (0.32) |
| *R*_free_^c^ | 0.24 (0.28) | 0.27 (0.39) |
| R.m.s. bond length (Å) | 0.006 | 0.007 |
| R.m.s. bond angle (°) | 0.925 | 1.573 |
| Average B value (Å^2^) (protein) | 85.09 | 57.64 |
| Average B value (Å^2^) (solvent) | 63.57 | 46.06 |
| Ramachandran plot |  |  |
| Favored (%) | 91.19 | 91.03 |
| Allowed (%) | 7.09 | 6.96 |
| Outliers (%) | 1.72 | 2.01 |

^a^ *R*_merge_ = ∑｜<I> - I｜/∑<I>.

^b^ *R*_cryst_ = ∑｜|Fo| - |Fc|｜/∑|Fo|.

^c^ *R*_free_ calculated with 5% of all reflections excluded from the refinement stages using high-resolution data.

The values in parentheses refer to the highest-resolution shells.

**Supplemental Table S3.** Primers used for site-directed mutagenesis

| **Mutants** | **Primer Sequence** |
| --- | --- |
| Y174F_F | CAT CGG TGG TGG ATT CAT TGC GTT TGA ATT CGC AC |
| Y174F_R | GTG CGA ATT CAA ACG CAA TGA ATC CAC CAC CGA TG |
| Y437F_F | CAA TGA TTT TCT CAT TCC CAA CAT TGG CAT CGG AC |
| Y437F_R | GTC CGA TGC CAA TGT TGG GAA TGA GAA AAT CAT TG |
| F. forward primer, R. reverse primer | |


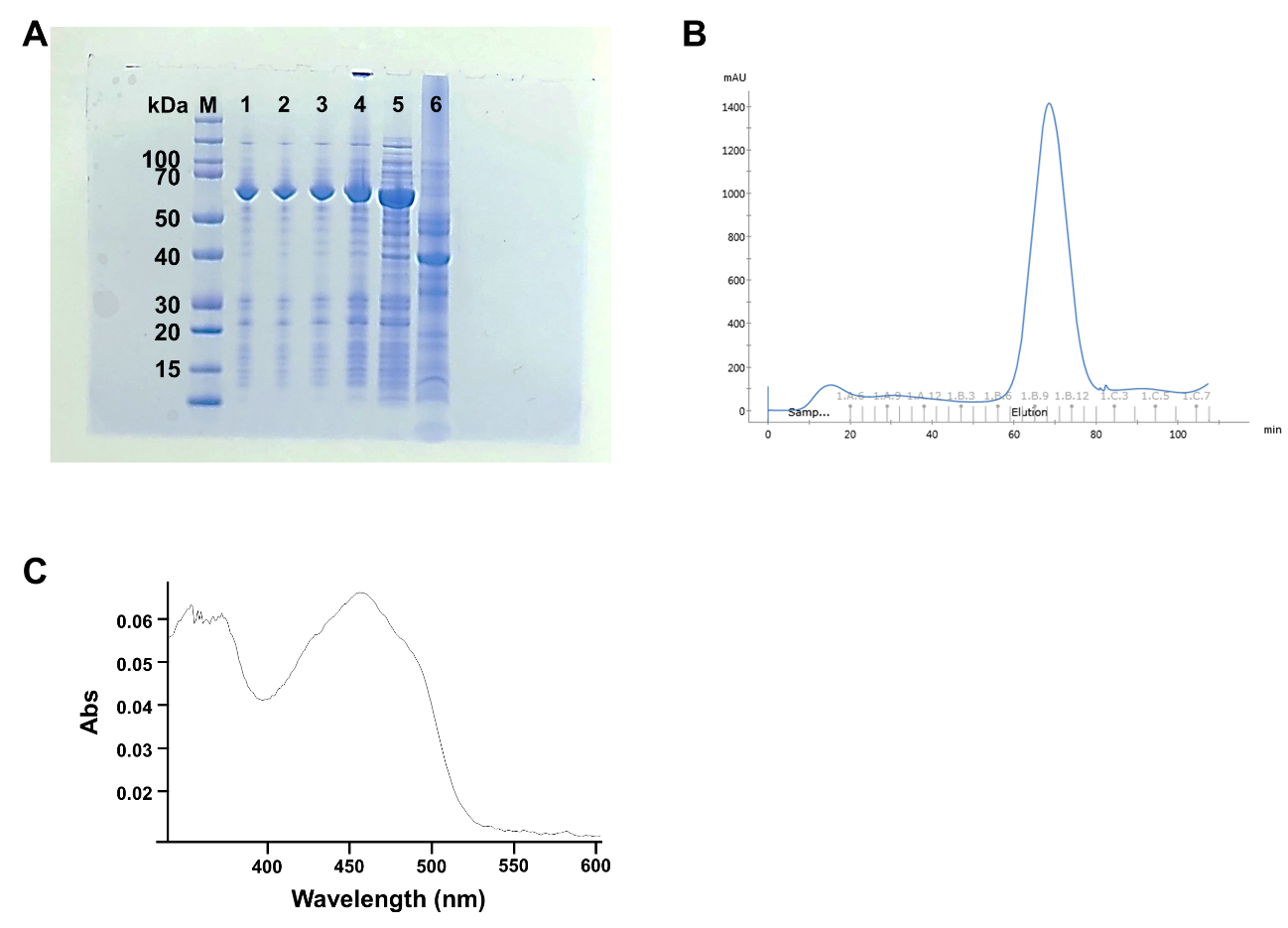


**Supplemental Figure S1.** Purification of *Gbs*MerA. (A) Sulfate–polyacrylamide gel electrophoresis (SDS–PAGE) analysis for *Gbs*MerA during purification. M marker, 1; 100mM imidazole elution fraction-first, 2; 100mM imidazole elution fraction-second, 3; 100mM imidazole elution fraction-third, 4; 250mM imidazole elution fraction, 5; soluble fraction, and 6; insoluble fraction. *Gbs*MerA was expressed using the pET32a(+) vector with a total 67kDa calculated molecular weight. (B) FPLC profile after purification using Superdex 200 10/300 GL column connected with ÄKTA Avant system (Cytiva, Marlborough, MA, USA). *Gbs*MerA was loaded onto an SEC column following enterokinase cleavage. (C). The UV-visible spectrum of *Gbs*MerA.

**
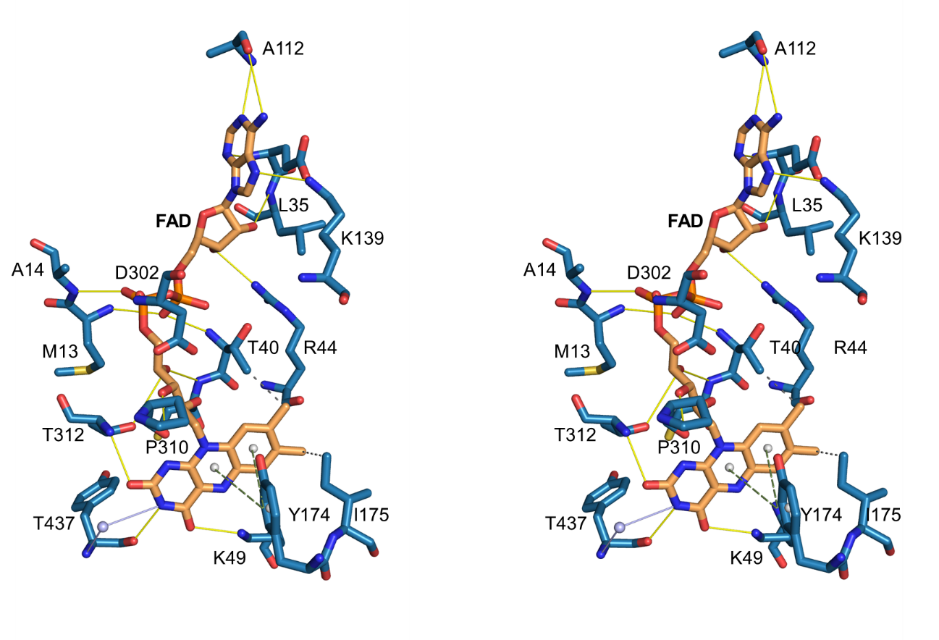
**

**Supplemental Figure S2.** Stereo view of the FAD binding site in the *Gbs*MerA monomer. The FAD molecule is indicated by an orange stick. The selected hydrogen bonding interactions are shown as yellow lines. stacking interactions (long dotted line) and hydrophobic interactions (short dotted line) are also indicated.


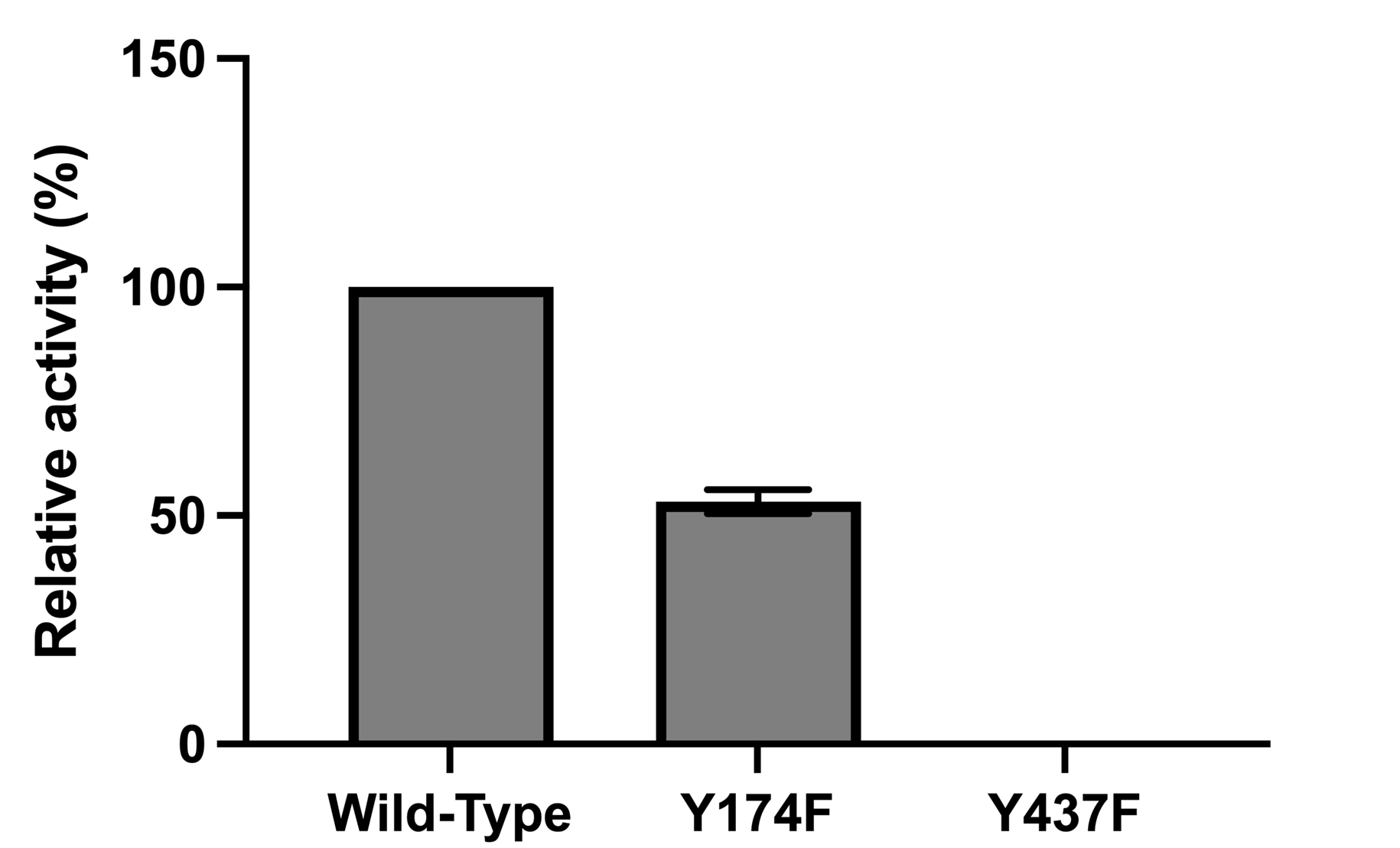


**Supplemental Figure S3.** Comparison of the activity of *Gbs*MerA with mutants. The error bars represent the standard error of triplicate experiments.
